# Marinopyrrole Derivatives as Potential Antibiotic Agents against Methicillin-Resistant *Staphylococcus aureus* (I)

**DOI:** 10.3390/md10040953

**Published:** 2012-04-24

**Authors:** Yan Liu, Nina M. Haste, Wdee Thienphrapa, Victor Nizet, Mary Hensler, Rongshi Li

**Affiliations:** 1 Department of Drug Discovery, H. Lee Moffitt Cancer Center and Research Institute, 12902 Magnolia Drive, Tampa, FL 33612, USA; Email: Yan.Liu@moffitt.org; 2 Skaggs School of Pharmacy and Pharmaceutical Sciences, University of California San Diego, La Jolla, CA 92093, USA; Email: nhaste@ucsd.edu (N.M.H.); vnizet@ucsd.edu (V.N.); 3 Department of Pediatrics, University of California San Diego, La Jolla, CA 92093, USA; Email: wthienph@ucsd.edu (W.T.); mhensler@ucsd.edu (M.H.)

**Keywords:** marinopyrrole, asymmetrical marinopyrroles, MRSA, antibiotics, SAR

## Abstract

Infections caused by drug-resistant pathogens are on the rise. The ongoing spread of methicillin-resistant *Staphylococcus aureus* (MRSA) strains exemplifies the urgent need for new antibiotics. The marine natural product, marinopyrrole A, was previously shown to have potent antibiotic activity against Gram-positive pathogens, including MRSA. However, its minimum inhibitory concentration (MIC) against MRSA was increased by >500 fold in the presence of 20% human serum, thus greatly limiting therapeutic potential. Here we report our discovery of a novel derivative of marinopyrrole A, designated **1a**, featuring a 2–4 fold improved MIC against MRSA and significantly less susceptibility to serum inhibition. Importantly, compound **1a** displayed rapid and concentration-dependent killing of MRSA. Compared to the natural product counterpart, compound **1a** provides an important natural product based scaffold for further Structure Activity Relationship (SAR) and optimization.

## 1. Introduction

**Chart 1:**
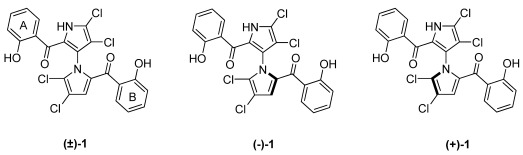
Chemical structures of marinopyrrole A.

Marinopyrroles were first reported to show antibiotic activity against methicillin-resistant *Staphylococcus aureus* (MRSA) several years ago [1]. Structural elucidation of the isolated marinopyrroles from an obligate marine *Streptomyces* (strain CNQ-418, collected from the sea floor near La Jolla, CA) by 2D NMR and X-ray crystallography revealed an unprecedented structure class. The densely halogenated 1,3-bipyrroles represented by marinopyrrole A (±)-**1** exist as enantiopure *M*-(−)-atropisomer, (−)-**1**, at ambient temperature (Chart 1). The enantiomer (+)-**1** was obtained after a chiral separation of synthetic (±)-**1** using chiral HPLC [2]. Due to their novel class of molecular structures and promising biological properties, the marinopyrroles have attracted considerable attention. In the last few years since the discovery of this uncommon bispyrrole structure of marine natural products, several synthetic and semi-synthetic efforts have successfully yielded a few series of novel marinopyrrole derivatives [2,3,4,5,6].

The global crisis of antibiotic resistance has spread rapidly over the last several decades. MRSA infections have reached epidemic proportions in many countries [7] and represent the most common cause of skin and soft tissue infections in the United States [8]. Both hospital-associated and community-associated MRSA can exhibit broader resistance to multiple classes of antibiotics [7,9,10,11]. Hospital-associated MRSA (HA-MRSA) infections are common among healthcare facilities and are resistant to many antibiotics. However, community-associated MRSA (CA-MRSA) strains are highly virulent and infect even healthy individuals; the incidence of these infections has skyrocketed in the last decade. The relative abandonment of antibiotic discovery and development by the pharmaceutical industry has created a vacuum in the introduction of new antibiotics to treat these increasingly problematic infections. Except for the addition of the oxazolidinone linezolid [12] in 2000, the lipopeptide daptomycin [13] in 2003, and FDA’s most recent approval of ceftaroline (Teflaro) [14] as an injectable antibiotic to treat adults with community acquired bacterial pneumonia (CABP) and acute bacterial skin and skin structure infections (ABSSSI) including MRSA, a very limited number of new antibiotics have been discovered over the last half a century, with only two new classes of anti-MRSA drugs having been approved in the last 40 years. Clearly, novel agents for the treatment of MRSA infections are urgently needed [15].

Recently, evaluation of the pharmacological properties has revealed that marinopyrrole A has potent concentration-dependent antibacterial activity against clinically-relevant hospital- and community-acquired MRSA strains, a prolonged post-antibiotic effect superior to the first-line current agents vancomycin and linezolid, and a favorable resistance profile [16]. Although marinopyrrole A (±)-**1**, (−)-**1** and (+)-**1** showed potent antibiotic activity [2] with limited toxicity to mammalian cell lines at >20× MIC, their antibiotic activity against MRSA were inhibited by 20% human serum [16]. Previous studies of synthetic or semi-synthetic marinopyrrole derivatives to date have yielded no new analogs that retained antibacterial activity in the presence of human serum [16], constituting a major challenge for therapeutic development. Here, we report our design and synthesis of novel marinopyrrole derivatives with excellent antibiotic activity against MRSA but only limited serum inactivation.

## 2. Results and Discussion

### 2.1. Synthesis and Structure Activity Relationships of Asymmetrical Marinopyrrole Derivatives

**Scheme 1:**
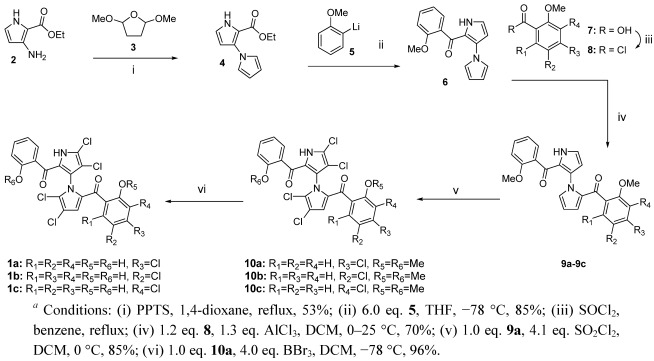
Synthesis of Novel Asymmetrical Marinopyrrole Derivatives *^a^*.

Two years ago, we reported the first total synthesis of marinopyrrole A along with “symmetrical” derivatives, (±)-**1** and 12 analogs, which bear the same substituents with the same substitution pattern on both phenyl ring A and B attached to the carbonyl groups (Chart 1) [6]. In addition to those symmetrical marinopyrrole derivatives, we envisage that the “asymmetrical” marinopyrrole derivatives shown in Scheme 1 should have different biological activity than their symmetrical counterparts. In particular, the molecules with diverse functional groups decorated on this unique 1,3-bipyrrole system may adopt specific conformations due to the restricted free rotation of the chiral axis. To this end, a series of novel asymmetrical marinopyrrole derivatives were designed and synthesized.

Chemistries to access both symmetrical and asymmetrical marinopyrrole derivatives were reported previously [2,6]. Briefly, 2-ethoxycarbonyl-3-aminopyrrole **2** was obtained using a published procedure [17]. A PPTS-promoted Clauson–Kaas reaction [2,18] between **2** and 2,5-dimethoxy-tetrahydrafuran **3** in dioxane under reflux gave rise to bispyrrole **4** in 53% yields. The mono-addition of lithiated anisole **5** to bispyrrole ester **4** in THF at −78 °C furnished mono acylated bispyrrole **6** in 85% yield [2]. Friedel–Crafts arylation of **6** with the acid chlorides **8**, generated *in situ* from the corresponding carboxylic acids **7** with thionyl chloride, afforded a series of marinopyrrole precursor **9a**–**9c**. A novel series of asymmetrical marinopyrrole derivatives **10a**–**10c** were obtained by tetrachlorination of the corresponding **9a**–**9c** using 4.1 equivalents of sulfuryl chloride (SO_2_Cl_2_) in DCM at 0 °C. Demethylation of **10a**–**10c** using BBr_3_ in DCM furnished **1a**–**1c** in >90% yield (Scheme 1) [2,6].

The anti-MRSA activity of the novel asymmetrical marinopyrroles shown in Scheme 1 was evaluated against a USA300 strain of community-associated MRSA. One of the derivatives, (4-chloro-2-hydroxyphenyl) (4,4′,5,5′-tetrachloro-2′-(2-hydroxybenzoyl)-1′*H*-1,3′-bipyrrol-2-yl)methanone (**1a**), exhibited potent antibacterial activity. The 2–4 fold improvement in antibacterial activity from the parent compound **1** was observed for this novel marinopyrrole derivative **1a** as shown in Table 1. The corresponding dimethoxy-congener, (4-chloro-2-methoxyphenyl)(4,4′,5,5′-tetrachloro-2′-(2-methoxybenzoyl)-1′*H*-1,3′-bipyrrol-2-yl)methanone, **10a**, was found to be inactive against MRSA at >200 μM. This observation is consistent with the result reported previously for compound **1** and its corresponding methoxy analog [2] as compound **1a** showed >1000 fold more potency than **10a**. This observed SAR was also supported by the current results from compounds **1b** and **1c**. Compound **1b**, with a chloro-substituent *ortho* to the hydroxyl group, was >128-fold more potent than **10a**, whereas derivative **1c** with chloro-substituent *para* to the hydroxyl group showed >64-fold more anti-MRSA activity than **10b**. Compound **1a** has the chloro-substituent *meta* to the hydroxyl group exhibiting 8–16-fold and 4–8-fold more anti-MRSA activity than **1c** and **1b**, respectively. The fact that the free hydroxyl groups are essential for the antibacterial activity suggested that the binding sites may contain both hydrogen bond donors and hydrogen bond receptors. Although the internal hydrogen bonding between the phenyl hydroxyl and adjacent carbonyl group was observed in an X-ray structure of (−)-**1** [1] and compounds **1a**–**1c** may behave similarly, this interaction can be weakened or interrupted if appropriate amino acid residues, such as Arg, His, Asn, Asp, Glu or Gln, are close by for interactions. Although thorough investigation is required to understand the observed structure activity relationships generated by compounds **1a**–**1c**, their physicochemical properties on the OR_5_ group in B ring due to the difference in p*K*_a_ values of their hydroxyl groups might, at least in part, play an essential role in addition to the different substitution pattern.

**Table 1:** Minimum inhibitory concentration (MIC) of Marinopyrrole Derivatives against methicillin-resistant *Staphylococcus aureus* (MRSA).

Compound	THB	THB + 20% Serum
**1 (parent)**	0.74	94–188
**10a**	>200	>200
**1a**	0.19–0.39	12.5–25
**1b**	1.56	>200
**1c**	3.13	>200

MIC in μM in Todd-Hewitt broth (THB) in the absence or presence of 20% human serum. All data were generated from experiments repeated four times.

Most significantly, not only did compound **1a** show increased antibacterial activity compared to the parent compound, marinopyrrole A (**1**), but its activity was also less inhibited upon the addition of 20% human serum (MIC 12.5–25 µM *vs.* 100–200 µM). In comparison to contemporary MRSA agents, compound **1a** is more potent than vancomycin and linezolid against USA300 MRSA strain TCH1516 [16].

### 2.2. *In Vitro* Time-Kill of Marinopyrrole Derivative ***1a***

*In vitro *time-kill studies were performed to characterize killing kinetics of derivative **1a** at 20×, 10× and 1× MIC and to compare them with that of the parent natural product marinopyrrole A (−)-**1** [16]. Derivative **1a** displayed strong concentration-dependent MRSA killing that was much more rapid than vancomycin or linezolid and was similar in profile to the parent compound [16]. The potency of derivative **1a** was especially evident at 20× the MIC (3.8 μM), yielding greater than a 4-log kill of MRSA at 4 h. This feature parallels the effects seen with the natural product parent compound (−)-**1**.

**Figure 1:**
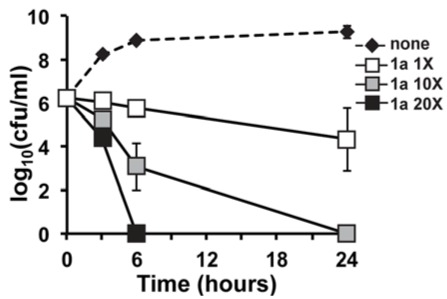
*In vitro* Time-kill Analysis for Marinopyrrole Derivative (**1a**) against the USA300 Community-associated MRSA Strain TCH1516. MRSA was subjected to increasing concentrations of 1× (0.39 μM), 10× (3.9 μM) and 20× (7.8 μM) the MIC of derivative **1a** or vehicle control (none). Derivative **1a** showed potent concentration-dependent *in vitro* bactericidal kinetics against MRSA at 10× (3.9 μM) and 20× (7.8 μM) MIC, along with bacteriostatic activity at 1× MIC (0.39 μM).

Compound (−)-**1** was also shown to have a favorable therapeutic index at 24 h in treated mammalian cells [16]. The natural product marinopyrrole A had a low propensity for toxicity at concentrations below 20× MIC. Upon closer examination, derivative **1a** displays more potent MRSA killing effects. For example, the tested concentration of **1a** (MIC 0.39 μM) was half of the concentration of the parent natural product tested in time-kill analyses (MIC 0.75 μM or 0.375 μg/mL) [16]. Secondly, at 3.9 μM (10× MIC) and 6 h incubation, **1a** causes 3-log decrease in bacterial survival (Figure 1). This improved upon the kinetics of the natural product, (−)-**1,** which generated only a two-log decrease in bacterial survival [16]. Furthermore, the actual tested compound concentration of (−)-**1** was 7.5 μM, a fold higher than that of derivative **1a** (3.9 μM) (Figure 1). Similar to (−)-**1**, this activity is much more rapid than vancomycin or linezolid [16]. In summary, we have discovered a novel natural product based derivative that retains the favorable and potent concentration-dependent bacterial killing characteristic of the natural product but at a lower concentration.

## 3. Experimental Section

### 3.1. Synthesis of Compounds ***4–10c***

All chemicals and solvents were purchased from commercial suppliers and used without further purification. Preparative flash column chromatography was performed on silica gel 60, 0.040–0.063 mm (EMD Chemicals). ^1^H NMR (400 MHz) spectra were recorded on a Varian AS400 with a 60-place automated sample changer. High resolution ESI-MS spectra were recorded on an Agilent ESI-TOF LC-MS 6200 system. Preparative HPLC was performed on a Gilson HPLC system with UV detectors and Gilson 215 liquid handler for auto injection and fraction collections (customized by HT Labs, San Diego, CA, USA). Analytical HPLC was performed on an Agilent 1100 series with diode array detectors and auto samplers. The specifications of HPLC analysis are as follows: flow rate, 1 mL/min; column, Intertsil, 2.5 μm, 4.6 × 150 mm; wavelength, 254 and 280 nm; mobile phase, A: H_2_O with 0.1% HCO_2_H, B: MeOH, gradient of 50–95% B in 25 min. All tested compounds possessed a purity of not less than 95%.

Ethyl 1′*H*-1,3′-bipyrrole-2′-carboxylate (**4**). To a stirred solution of 2,5-dimethoxy-tetrahydrofuran **3** (1.6 mL, 12.4 mmol, 1.3 equiv) and PPTS (3.2 g, 12.4 mmol, 1.3 equiv) in 1,4-dioxane (20 mL) at 50 °C, a solution of aminopyrrole **2** (1.47 g, 9.55 mmol, 1.0 eq.) in 1,4-dioxane (6 mL) was added slowly. The resulting mixture was brought to reflux and stirred for 1 h. The reaction mixture was then concentrated, dissolved in EtOAc (50 mL) and dried over anhydrous MgSO_4_. The crude product was purified by flash column chromatography (10% EtOAc in hexane) to give **4** as a yellow solid (1.036 g, 53% yield). ^1^H NMR (400 MHz, CDCl_3_) δ 8.99 (s, 1H), 7.04–7.01 (m, 2H), 6.89 (t, *J* = 3.1 Hz, 1H), 6.29 (t, *J* = 2.9 Hz, 1H), 6.27–6.24 (m, 2H), 4.28 (q, *J* = 7.1 Hz, 2H), 1.29 (t, *J* = 7.1 Hz, 3H).

1′*H*-1,3′-Bipyrrol-2′-yl(2-methoxyphenyl)methanone (**6**). To a solution of 2-bromoanisole (1.39 g, 7.43 mmol, 5.63 equiv) in anhydrous THF (5 mL) at −78 °C, a 2.5M solution of BuLi in hexane (3 mL, 7.50 mmol, 5.68 eq.) was added dropwise, the resulting mixture was stirred for 1 h. at −78 °C to generate the lithiated anisole. In an oven-dried flask, compound **4** (270 mg, 1.32 mmol, 1 eq.) was dissolved in THF (5 mL) and cooled to −78 °C, the lithiated anisole **5** was transferred into the solution containing **4** dropwise via syringe, and the resulting mixture was stirred for 1 h. at −78 °C. To the reaction mixture, an aqueous solution of NH_4_Cl (5 mL) was added, the mixture was extracted with EtOAc, dried over anhydrous Na_2_SO_4_. The crude product was purified by flash column chromatography (10% EtOAc in hexane) to give **6** as a light brown solid (0.30 g, 85% yield). ^1^H NMR (400 MHz, CDCl_3_) δ 9.35 (s, 1H), 7.28–7.22 (m, 1H), 7.20 (dd, *J* = 7.5, 1.7 Hz, 1H), 7.02 (t, *J* = 3.0 Hz, 1H), 6.80 (td, *J* = 7.5, 0.9 Hz, 1H), 6.68 (d, *J* = 8.4 Hz, 1H), 6.48–6.43 (m, 2H), 6.27 (t, *J* = 2.8 Hz, 1H), 5.88 (m, 2H), 3.63 (s, 3H).

(2-(4-Chloro-2-methoxybenzoyl)-1′H-1,3′-bipyrrol-2′-yl)(2-methoxyphenyl)methanone (**9a**). Into a solution of 4-chloro-2-methoxybenzoic acid (125 mg, 0.67 mmol, 1.2 eq.) in benzene (1.5 mL), SOCl_2_ (1.5 mL) was added at room temperature and the resulting solution was refluxed for 2 h. The reaction mixture was concentrated under vacuum to generate 4-chloro-2-methoxybenzoyl chloride **8a** which was used directly in the next step without purification. A solution of **8a** in CH_2_Cl_2_ (DCM) (2 mL) was added to a slurry of AlCl_3_ (96 mg, 1.3 eq.) in DCM (2.5 mL) at 0 °C and then a solution of **6** (150 mg, 0.56 mmol, 1.0 equiv) in DCM (1.5 mL) was added dropwise. The resulting solution was allowed to warm to room temperature and stirred overnight. A saturated solution of NaHCO_3_ (10 mL) and DCM (10 mL) was then added and the resulting mixture was stirred for 1 h, and then filtered though Celite^®^. The mixture was extracted with DCM (2 × 10 mL), the organic layer was dried over anhydrous Na_2_SO_4_, and purified by flash column chromatography (hexanes:EtOAc) to afford 172 mg of **9a** as a white solid, 70% yield. ^1^H NMR (400 MHz, CDCl_3_) δ 9.83 (s, 1H), 7.22–7.14 (m, 2H), 7.12 (d, *J* = 7.8 Hz, 1H), 7.04 (t, *J* = 3.0 Hz, 1H), 6.95–6.88 (m, 2H), 6.73–6.63 (m, 3H), 6.31 (dd, *J* = 4.0, 1.7 Hz, 1H), 6.27 (t, *J* = 2.8 Hz, 1H), 5.83 (dd, *J* = 4.0, 2.6 Hz, 1H), 3.77 (s, 3H), 3.69 (s, 3H).

(2-(5-Chloro-2-methoxybenzoyl)-1′*H*-1,3′-bipyrrol-2′-yl)(2-methoxyphenyl)methanone (**9b**). 5-Chloro-2-methoxybenzoyl chloride (**8b**) was generated *in situ* from the corresponding acid using the same method as **8a**. Compound **9b** (88 mg, 67% yield) was obtained using the same method as **9a** described above. ^1^H NMR (400 MHz, CDCL_3_) δ 9.78 (s, 1H), 7.31 (dd, *J* = 8.8, 2.7 Hz, 1H), 7.25–7.15 (m, 2H), 7.06 (dd, *J* = 4.3, 1.7 Hz, 2H), 6.85 (d, *J* = 8.9 Hz, 1H), 6.77–6.66 (m, 3H), 6.32 (dd, *J* = 4.0, 1.7 Hz, 1H), 6.30 (t, *J* = 2.8 Hz, 1H), 5.87 (dd, *J* = 4.0, 2.6 Hz, 1H), 3.75 (s, 3H), 3.69 (s, 3H).

(2-(3-Chloro-2-methoxybenzoyl)-1′*H*-1,3′-bipyrrol-2′-yl)(2-methoxyphenyl)methanone (**9c**). 3-Chloro-2-methoxybenzoyl chloride (**8c**) was generated *in situ* from the corresponding acid using the same method as **8a**. Compound **9c** (90 mg, 68% yield) was obtained using the same method as **9a** described above. ^1^H NMR (400 MHz, CDCL_3_) δ 9.81 (s, 1H), 7.52 (dd, *J* = 7.8, 1.6 Hz, 1H), 7.39 (dd, *J* = 8.0, 1.6 Hz, 1H), 7.24–7.19 (m, 1H), 7.10 (dt, *J* = 5.7, 2.2 Hz, 2H), 6.87 (dd, *J* = 2.6, 1.6 Hz, 1H), 6.79 (d, *J* = 7.9 Hz, 1H), 6.66–6.59 (m, 3H), 6.31 (t, *J* = 2.8 Hz, 1H), 6.05 (dd, *J* = 4.1, 2.6 Hz, 1H), 3.57 (s, 3H), 3.52 (s, 3H).

(4-Chloro-2-methoxyphenyl)(4,4′,5,5′-tetrachloro-2′-(2-methoxybenzoyl)-1′*H*-[1,3′-bipyrrole]-2-yl)methanone (**10a**). To a solution of compound **9a** (82 mg, 0.19 mmol, 1 eq.) in DCM (5 mL) at 0 °C, SO_2_Cl_2_ (64 μL, 0.78 mmol, 4.1 equiv) was added dropwise, and the solution was allowed to stir at 0 °C for 1 h. Saturated aqueous NaHCO_3_ solution (2 mL) was added and the resulting mixture was extracted with DCM (3 × 4 mL). The combined organic layers were dried with anhydrous MgSO_4_, filtered, and concentrated. The residue was purified by flash column chromatography (silica gel, hexane:EtOAc 4:1) to afford **10a** (92 mg, 85% yield) as an off-white solid. ^1^H NMR (400 MHz, CDCl_3_) δ 10.38 (s, 1H), 7.25–7.21 (m, 2H), 7.13 (d, *J* = 7.9 Hz, 1H), 6.96–6.94 (m, 2H), 6.76 (d, *J* = 8.3 Hz, 1H), 6.68 (t, *J* = 7.8 Hz, 1H), 6.31 (s, 1H), 3.80 (s, 3H), 3.72 (s, 3H); HRMS (ESI-TOF) [M + H]^+^ calcd for C_24_H_16_Cl_5_N_2_O_4_ 570.9547, found 570.9537; HPLC purity, 95.2%.

(5-Chloro-2-methoxyphenyl)(4,4′,5,5′-tetrachloro-2′-(2-methoxybenzoyl)-1′*H*-1,3′-bipyrrol-2-yl)methanone (**10b**). The same procedure described for **10a** was used to synthesize **10b** (87 mg, 80% yield). ^1^H NMR (400 MHz, CDCL_3_) δ 10.28 (s, 1H), 7.36 (dd, *J* = 8.9, 2.7 Hz, 1H), 7.31–7.27 (m, 1H), 7.19 (dd, *J* = 7.5, 1.7 Hz, 1H), 7.07 (d, *J* = 2.6 Hz, 1H), 6.88 (d, *J* = 8.9 Hz, 1H), 6.79 (d, *J* = 8.4 Hz, 1H), 6.73 (td, *J* = 7.5, 0.8 Hz, 1H), 6.33 (s, 1H), 3.78 (s, 3H), 3.73 (s, 3H).

(3-Chloro-2-methoxyphenyl)(4,4′,5,5′-tetrachloro-2′-(2-methoxybenzoyl)-1′*H*-1,3′-bipyrrol-2-yl)methanone (**10c**). The same procedure described for **10a** was used to synthesize **10c** (80 mg, 74%). ^1^H NMR (400 MHz, CDCL_3_) δ 10.30 (s, 1H), 7.57 (dd, *J* = 7.9, 1.5 Hz, 1H), 7.37–7.27 (m, 2H), 7.11 (dd, *J* = 7.5, 1.7 Hz, 1H), 6.85 (t, *J* = 7.9 Hz, 1H), 6.76 (d, *J* = 8.4 Hz, 1H), 6.67 (t, *J* = 7.5 Hz, 1H), 6.57 (s, 1H), 3.83 (s, 3H), 3.73 (s, 3H).

### 3.2. Synthesis of Compounds ***1a–1c***

(4-Chloro-2-hydroxyphenyl)(4,4′,5,5′-tetrachloro-2′-(2-hydroxybenzoyl)-1′*H*-1,3′-bipyrrol-2-yl)methanone (**1a**). To a solution of **10a** (33 mg, 0.058 mmol) in anhydrous DCM (1 mL) was slowly added 1.0 M solution of BBr_3_ in DCM (23 μL, 0.23 mmol, 4 eq.) via a syringe under N_2_ at −78 °C. After being stirred for 0.5 h, the mixture was quenched by addition of MeOH (0.5 mL) and extracted with DCM (3 × 10 mL). The combined organic layers were dried over anhydrous MgSO_4_, filtered and concentrated in vacuum. The residue was purified by column chromatography (silica gel, hexanes:12% EtOAc) to give **1a** (30 mg, 96% yield) as a yellow solid. ^1^H NMR (400 MHz, CDCl_3_) δ 11.34 (s, 1H), 10.39 (s, 1H), 9.77 (s, 1H), 7.47 (d, *J* = 8.6 Hz, 1H), 7.43 (dd, *J* = 8.0, 1.7 Hz, 1H), 7.36 (ddd, *J* = 8.8, 7.4, 1.6 Hz, 1H), 7.04 (d, *J* = 2.0 Hz, 1H), 6.95–6.90 (m, 1H), 6.87 (dd, *J* = 8.6, 2.0 Hz, 1H), 6.68 (s, 1H), 6.53 (ddd, *J* = 8.0, 7.3, 1.1 Hz, 1H); HRMS (ESI-TOF) [M + H]^+^ calcd for C_22_H_12_Cl_5_N_2_O_4_ 542.9234, found 542.9237; HPLC purity 96.6%.

(5-Chloro-2-hydroxyphenyl)(4,4′,5,5′-tetrachloro-2′-(2-hydroxybenzoyl)-1′*H*-1,3′-bipyrrol-2-yl)methanone (**1b**). The same procedure as **1a** was followed to obtain **1b** (28 mg, 90%) from **10b** (31 mg, 0.055 mmol). ^1^H NMR (400 MHz, CDCL_3_) δ 11.10 (s, 1H), 10.33 (s, 1H), 9.89 (s, 1H), 8.06 (dd, *J* = 7.9, 1.8 Hz, 1H), 7.95 (d, *J* = 2.4 Hz, 1H), 7.83 (d, *J* = 8.3 Hz, 1H), 7.71–7.64 (m, 1H), 7.49–7.41 (m, 2H), 6.97 (dd, *J* = 8.9, 2.1 Hz, 1H), 6.87 (s, 1H); HRMS (ESI-TOF) [M + Na]^+^ calcd for C_22_H_11_Cl_5_N_2_O_4_Na 564.9054, found 564.9055; HPLC purity, 95.0%.

(3-Chloro-2-hydroxyphenyl)(4,4′,5,5′-tetrachloro-2′-(2-hydroxybenzoyl)-1′*H*-1,3′-bipyrrol-2-yl)methanone (**1c**)**.** The same procedure as **1a** was followed to obtain **1c** (26 mg, 91%) from **10c** (29 mg, 0.05mmol). ^1^H NMR (400 MHz, CDCL_3_) δ 11.74 (s, 1H), 10.48 (s, 1H), 9.93 (s, 1H), 8.06 (d, *J* = 7.9 Hz, 1H), 7.94 (d, *J* = 8.0 Hz, 1H), 7.82 (d, *J* = 8.2 Hz, 1H), 7.67 (t, *J* = 7.8 Hz, 1H), 7.61 (d, *J* = 7.9 Hz, 1H), 7.45 (t, *J* = 7.6 Hz, 1H), 6.96 (t, *J* = 7.9 Hz, 1H), 6.83 (s, 1H); HRMS (ESI-TOF) [M + Na]^+^ calcd for C_22_H_11_Cl_5_N_2_O_4_Na 564.9054, found 564.9058; HPLC purity, 97.4%.

### 3.3. *In Vitro* Antibacterial Assays

TCH1516, a USA300 strain of community-associated MRSA, was obtained from the American Type Culture Collection and used for biological assays. MICs were determined by broth microdilution according to Clinical and Laboratory Standards Institute guidelines except that Todd-Hewitt broth (THB) was used in place of Mueller-Hinton broth. Assays were done in 96-well tissue-culture treated plates. Vancomycin served as a control antibiotic. MIC assays in 20% human serum were assessed by bacterial metabolic activity in resazurin as described [16].

### 3.4. *In Vitro* Time-Kill Analysis

The bactericidal activity of derivative **1a** against the CA-MRSA isolate TCH1516 were assessed by time-kill analysis essentially as described previously [16,19,20,21]. Briefly, MRSA was grown overnight in Todd-Hewitt Broth (THB) at 37 °C with shaking. Following overnight growth, MRSA was inoculated in fresh media for growth to mid-logarithmic phase. At the start of the time-kill assay, bacteria (starting inoculum ~5 × 10^5^ cfu/mL) were added to duplicate 5 mL polystyrene round-bottom tubes (Falcon, Bedford, MA, USA) containing 20×, 10×, 1×, none of the MIC of derivative **1a** (0.39 μM) or an equivalent amount of DMSO vehicle control (Figure 1, none). These cultures were incubated in a shaking 37 °C incubator for 24 h. To determine the rate of antibiotic killing, small aliquots were removed from tubes at 0, 3, 6 and 24 h and serially diluted for cfu enumeration on THA plates. The limit of detection for the time-kill assay was 1.6 (log_10_ cfu/mL).

## 4. Conclusions

In summary, we designed and synthesized a novel series of asymmetrical derivatives of marine natural product, marinopyrrole A, and discovered that a novel derivative, **1a** (MIC = 0.19–0.39 μM), exhibits significantly greater activity than linezolid and vancomycin against MRSA. Moreover, *in vitro* time-kill kinetics of **1a** demonstrated potent concentration-dependent killing. These qualities were similar to those displayed by the parent natural product in time-kill studies, but **1a** was 2–4-fold more potent at lower compound concentrations. One of the main drawbacks of the natural product has been its significant inhibition in the presence of human serum. For example, the antibiotic activity of (−)-**1** was inactivated (MIC increased 256–522×) in the presence of serum [16]. Here, **1a** again exhibits an advantage, with only 32–64 fold increase in MIC in 20% human serum (Table 1). Further derivatization and SAR optimization will aim to engineer out the propensity for serum inhibition. Design and synthesis of both symmetrical and asymmetrical marinopyrrole derivatives are actively ongoing. The optimization of their structure and activity for use as potential antibiotic agents against MRSA will be reported in due course.
